# A systematic review of transfusion-transmitted malaria in non-endemic areas

**DOI:** 10.1186/s12936-018-2181-0

**Published:** 2018-01-16

**Authors:** Federica Verra, Andrea Angheben, Elisa Martello, Giovanni Giorli, Francesca Perandin, Zeno Bisoffi

**Affiliations:** 1 Centre for Tropical Diseases, Sacro Cuore-Don Calabria Hospital, 37024 Negrar, Verona Italy; 2 Department of Veterinary Sciences, University of Turin, Grugliasco, 10095 Turin, Italy

**Keywords:** Blood transfusion, Malaria, *Plasmodium*, Blood component transfusion, Transfusion-transmitted malaria (TTM)

## Abstract

**Background:**

Transfusion-transmitted malaria (TTM) is an accidental *Plasmodium* infection caused by whole blood or a blood component transfusion from a malaria infected donor to a recipient. Infected blood transfusions directly release malaria parasites in the recipient’s bloodstream triggering the development of high risk complications, and potentially leading to a fatal outcome especially in individuals with no previous exposure to malaria or in immuno-compromised patients. A systematic review was conducted on TTM case reports in non-endemic areas to describe the epidemiological characteristics of blood donors and recipients.

**Methods:**

Relevant articles were retrieved from Pubmed, EMBASE, Scopus, and LILACS. From each selected study the following data were extracted: study area, gender and age of blood donor and recipient, blood component associated with TTM, *Plasmodium* species, malaria diagnostic method employed, blood donor screening method, incubation period between the infected transfusion and the onset of clinical symptoms in the recipient, time elapsed between the clinical symptoms and the diagnosis of malaria, infection outcome, country of origin of the blood donor and time of the last potential malaria exposure.

**Results:**

*Plasmodium* species were detected in 100 TTM case reports with a different frequency: 45% *Plasmodium falciparum,* 30% *Plasmodium malariae,* 16*% Plasmodium vivax,* 4*% Plasmodium ovale,* 2% *Plasmodium knowlesi,* 1% mixed infection *P. falciparum/P. malariae*. The majority of fatal outcomes (11/45) was caused by *P. falciparum* whilst the other fatalities occurred in individuals infected by *P. malariae* (2/30) and *P. ovale* (1/4). However, non *P. falciparum* fatalities were not attributed directly to malaria. The incubation time for all *Plasmodium* species TTM case reports was longer than what expected in natural infections. This difference was statistically significant for *P. malariae* (*p* = 0.006). A longer incubation time in the recipient together with a chronic infection at low parasite density of the donor makes *P. malariae* a subtle but not negligible risk for blood safety aside from *P. falciparum*.

**Conclusions:**

TTM risk needs to be taken into account in order to enhance the safety of the blood supply chain from donors to recipients by means of appropriate diagnostic tools.

## Background

Malaria is an infectious disease caused by intracellular protozoan parasites of the genus *Plasmodium* responsible for a potentially fatal acute febrile illness following invasion and multiplication in human red blood cells (RBCs) during their complex life cycle. Five species of *Plasmodium* are currently known to cause malaria in humans: the deadliest *Plasmodium falciparum* and *Plasmodium vivax*, *Plasmodium malariae*, *Plasmodium ovale*, *Plasmodium knowlesi*. Malaria parasites are naturally transmitted by the infective bites of female *Anopheles* mosquitoes during their blood meal. Malaria can manifest with severe symptoms leading to a fatal outcome in non-immune individuals, often young children and pregnant women in endemic areas or naïve adults in non-endemic settings, and remains asymptomatic in adults who have acquired a premunition maintained by repeated antigen exposure.

Transfusion-transmitted malaria (TTM) is an accidental *Plasmodium* infection caused by the transfusion of whole blood or a blood component from a malaria infected donor to a recipient, described for the first time by Woolsey in 1911 [1], that may cause severe clinical symptoms in the recipients, especially in those with no previous exposure to malaria or in immuno-compromised patients due to other coexisting diseases. Several systematic reviews have addressed the knowledge gap still existing in the epidemiology of TTM in the United States [2], Canada [3] and the Americas [4].

*Plasmodium falciparum*, *P. vivax* and *P. malariae* are the species most frequently detected in TTM [5]. Various aspects of the parasite biology make this accidental route of infection feasible such as the persistence of infection: *P. falciparum* can persist for at least 1 year before being cleared, *P. vivax* for 3 years whereas *P. malariae* is known to remain as a chronic infection at low density for decades [6]. All *Plasmodium* species are able to survive in stored blood, even if frozen, and retain their viability for at least 1 week, possibly well over 10 days depending on the conditions of storage; in fact, microscopically detectable malaria parasites were present even after 28 days of storage at 4 °C although a decrease of infectivity after 2 weeks was observed [6, 7]. An important difference between the natural infection and TTM is that the former undergoes an initial asymptomatic phase (pre-erythrocytic) which allows the activation of innate immunity cells against malaria parasites. This early phase has advantages on both sides of the host parasite arms race: the innate immunity gives the naïve host time to develop a more specific protective immunity; meanwhile the parasites manipulate the host’s immune system in order to escape. Infected blood transfusions directly release malaria parasites in the recipient’s bloodstream triggering the development of high risk complications and potentially leading to a fatal outcome [8]. Experimental evidence suggests that as few as 10 infected RBCs can be sufficient to transmit the infection; thus, even a small inoculum is potentially infectious. However, the mean incubation period for TTM is generally longer than the mean incubation period for the mosquito-transmitted malaria (MTM) for all *Plasmodium* species as reported by [9]: 16.0 (8–29) days for *P. falciparum* TTM compared to 13.1 (7–27) days in *P. falciparum* MTM; 57. 2 (6–106) days for *P. malariae* TTM compared to 34.8 (27–37) days for *P. malariae* MTM; 19.6 (8–30) days for *P. vivax* TTM compared to 13.4 (8–31) days for *P. vivax* MTM; 23 days for *P. ovale* TTM compared to 13.6 (11–16) days for *P. ovale* MTM [9]. Blood components such as RBCs, platelets and plasma, are commonly transfused to treat various conditions ranging from surgical procedures causing a temporary anaemia to a chronic one due to haematological disorders (haemoglobinopathies, glucose-6-phosphate-dehydrogenase (G6PD) deficiency, haemophilia). Blood banks require a preliminary screening of a potential blood donor to exclude the risk of current or previous infections which can be transmitted by a blood transfusion, including malaria. Criteria for haemovigilance are defined by the World Health Organization (WHO) and are adapted to each country according to national guidelines. Some countries such as USA rely on a pre-donation questionnaire for the screening of potential infected donors whereas some others, including France, UK and Australia, use antibody testing on donors who are considered at risk on the basis of the preliminary questionnaire [3]. Appropriate diagnostic tools need to be employed in order to enhance the safety of the blood supply chain from donors to recipients tailored to the local TTM risk. The sensitivity and specificity of the screening strategy of blood donors remains the crucial issue in order to ensure the safety of blood transfusions particularly in the case of malaria: in fact, serological tests currently employed do not indicate the actual parasitaemia because antibody levels can remain elevated for many years after infection of *P. falciparum* and *P. vivax* [10]. Also, the initial clinical symptoms are generally aspecific making the diagnosis more difficult and resulting in a further delay. Delayed or missed diagnosis of *P. falciparum* in particular increases the risk of severe disease which may be fatal especially in non-immune individuals.

Furthermore new technologies are available to selectively inactivate pathogens without damaging cells or plasma; a combination of riboflavin as a photosensitizer with a UV light illumination device (Mirasol System for Whole Blood; Terumo BCT, Lakewood, Colo.) proved to substantially reduce *P. falciparum* infectivity in whole blood samples without altering cell quality parameters [11]; this inactivation technology may well represent another layer of control to reduce the risk of TTM.

Lastly, infected recipients who do not develop any clinical illness may become asymptomatic carriers and thus a reservoir of malaria parasites if competent vectors were to be present; this event has serious implications especially in non-endemic countries where the majority of the population has never been exposed to malaria parasites.

The primary objective of this systematic review was to describe the epidemiological characteristics of TTM in non-endemic countries based on data available in the literature in order to evaluate the extent and dynamics of this particular risk of malaria transmission. The review specifically investigated: (i) which *Plasmodium* species are more often detected in TTM; (ii) if other *Plasmodium* species besides *P. falciparum* are likely to cause a lethal outcome of TTM; (iii) whether the incubation time in TTM is longer than in the natural infection; (iv) which blood component is more likely to be infective for the recipient (whole blood, red blood cells, platelets or plasma); (v) which diagnostic methods were used in donor screening and recipient diagnosis (microscopy, serological or molecular tests).

## Methods

### Literature search

A systematic review of all articles on TTM in non-endemic areas was carried out. Relevant articles were retrieved from Pubmed, EMBASE, Scopus, and LILACS databases using combinations of the following search terms: “malaria”, “blood transfusion”, “*Plasmodium*”, “transfusion”, adapted to each database without date or language restrictions until May 17th 2017. TTM cases in USA were retrieved from the annual Morbidity and Mortality Weekly Reports (MMWR) malaria surveillance reports. The following combination of MeSH and free string terms were used specifically in Pubmed:

((“Platelet Transfusion”[MeSH] OR “Transfusion Medicine”[MeSH] OR “Lymphocyte Transfusion”[MeSH] OR “Leukocyte Transfusion”[MeSH] OR “Erythrocyte Transfusion”[MeSH] OR “Blood Component Transfusion”[MeSH]) OR (Transfusion*)) AND ((malaria*) OR (Plasmodi*) OR (malaria [MeSH])). Original research papers were included and additional references retrieved from narrative reviews; restriction to case reports was deemed necessary as the main scope of this systematic review was to investigate in fine details the relevant characteristics of each reported case of TTM. Two independent investigators (FV, EM) screened titles and abstracts, selected articles for full text review, performed the final article selection; a third reviewer (AA) was consulted in case of disagreement in order to reach a consensus. Case reports were excluded if the *Plasmodium* species was described as “tertian” without further identification. Also, case reports occurred in malaria endemic countries were not considered unless the case report was ascertained to have happened in a non-endemic area of the country. Articles in Chinese, Russian, Arabic and Turkish languages without at least a summary in English were dropped. From each study the following data was extracted: study area, gender and age of blood donor and recipient, blood component transfused, *Plasmodium* species, malaria diagnostic method employed, blood donor screening method, incubation period (i.e. the time elapsed between the infected transfusion and the onset of clinical symptoms in the recipient), delayed diagnosis (i.e. time elapsed between the onset of clinical symptoms and the diagnosis of malaria), infection outcome, country of origin of the blood donor and time of the last potential malaria exposure. The protocol for this systematic review was published on PROSPERO database with the registration number CRD 42017062298.

### Statistical analysis

The incubation time of each TTM case report was analysed through standard one sample two-tailed t-tests (level of significance α = 0.05) to evaluate the difference between incubation periods of TTM and MTM for each *Plasmodium* species. Reference mean values of MTM were drawn from the results shown by Dover and Schultz [9]. All statistical analyses were performed using R software, version 3.3.3 [12].

## Results

**Fig. 1:**
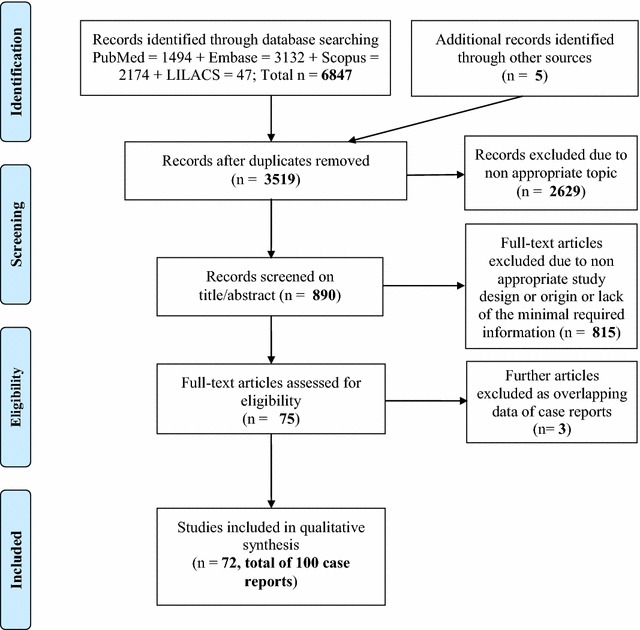
Flow diagram of the articles selection on transfusion transmitted- malaria in non-endemic areas

The number of selected papers at each step of the screening and criteria for exclusion/inclusion are reported in the flow diagram (Fig. 1); 100 case reports of TTM were retrieved for the purpose of this review and the main epidemiological data is provided by Table 1. Fifty-four of these case reports occurred in the American continent, 38 in Europe, 3 in the Mediterranean area, 1 in India, 4 in South-East Asia.

**Table 1:** Reported cases of transfusion- transmitted malaria (TTM) in malaria non-endemic areas

Country^a^	Year	Donor gender and age	Donor origin and last exposure	Recipient gender and age	Recipient incubation (delayed diagnosis)	Recipient outcome	Blood component transfused	*Plasmodium* species	Diagnosis method recipient (donor)	References
*Canada*										
Western region	1936	M	Mediterranean area	F 13 years	3 weeks (5 weeks)	Recovery	WB	*P. malariae*	LM (LM)	[17]
Ontario	1936	M	Romania 25 years	F	26 days (2 weeks)	Recovery	WB	*P. malariae*	LM (LM)	[18]
Alberta	1977	F 23 years	Africa 2 years	F 60 years	29 days (29 days)	Recovery	WB	*P. ovale*	LM (IFAT)	[19]
Quebec	1994	N/A	Cameroon > 3 years	M 63 years	N/A (3 weeks)	Recovery	RBCs, PLTs, FFP	*P. falciparum*	LM (LM)	[20]
Ontario	1995	M	Mali 4 years	F 24 years	15 days (3 days)	Recovery	RBCs	*P. falciparum*	LM, PCR (PCR)	[20]
Ontario	1997	F 19 years	Ghana 4 years	F 62 years	21 days (5 weeks)	Recovery	RBCs, FFP	*P. falciparum*	LM, PCR	[20]
*USA*										
New York	1911	N/A	N/A	M 54 years	11 days	“Pernicious anaemia”	WB	*P. vivax*	LM	[1]
Colorado	1929	M 32 years	Greece 16 years	F 2½ years	19–25 days (on the day)	Recovery	WB	*P. malariae*	LM (LM)	[21]
New York	1932	M	Italy	F 1.5 years	4 weeks (17 days)	Recovery	WB	*P. malariae*	LM	[22]
New York	1932	M	Italy 12 years	M 9 months	6 weeks	Recovery	WB	*P. malariae*	LM	[22]
New York	1933	F	Greece	F 8 years	< 8 weeks	Death due to pneumonia	WB	*P. malariae*	LM	[22]
New York	1936	M	Greece 33 years	F 1 year	29 days	Recovery	WB	*P. malariae*	LM	[22]
New York	1936	M	Colombia 10 years	M 3 years	2 months	Recovery	WB	*P. malariae*	LM	[22]
New York	1944	M 40 years	North Africa veteran 1 year	F 32 years	11–4 days (35 days)	Recovery	WB	*P. malariae*	LM (LM)	[23]
Rhode Island	1946	M or F	Italy or New England	F 40 years	2 months (2 days)	Recovery	WB, FFP	*P. malariae*	LM (LM)	[24]
Pennsylvania	1946	N/A	Army returnee	F	3 weeks (9 days)	Recovery	WB	*P. vivax*	LM	[25]
California	1968	M 19 years	Vietnam veteran 7 months	M 60 years	4 days (on the day)	N/A	WB	*P. falciparum*	LM (LM)	[26]
Connecticut	1968	N/A	Mexico 5 years	F 8 months	6 ½ months	Recovery	WB	*P. malariae*	LM	[27]
Washington state	1968	M 22 years	Vietnam veteran 1 year	F 54 years	13 days (9 days)	Recovery	WB	*P. falciparum*	LM (LM, IFAT)	[28]
Oklahoma	1968	M 21 years	Vietnam veteran 5 months	F 25 years	16 days (7 days)	Recovery	WB	*P. falciparum*	LM (LM)	[28]
Washington D.C.	1969	M	Nigeria > 2 years	M 56 years	17 days (6 days)	Death	WB	*P. falciparum*	LM (LM, IFAT)	[29]
New York	1970	M	Ghana 1 year	M 34 years	6 days (2 days)	Recovery	WB	*P. falciparum*	LM (LM, IFAT)	[30]
Chicago	1972	N/A	N/A	M 5 months	16 days (6 days)	Recovery	WB	*P. vivax*	LM	[31]
New York	1971	N/A	N/A	M 24 years	Multiple transfusions (7 days)	Recovery	WB	*P. vivax*	LM (IFAT)	[32]
New York	1974	N/A	N/A	F 42 years	9 weeks (on the day)	Recovery	RBCs, PLTs, FFP	*P. malariae*	LM (IFAT)	[32]
New York	1974	F 53 years	Greece 22 years	F 78 years	Multiple transfusions (~ 30 days)	Recovery	WB, RBCs	*P. malariae*	LM, IFAT (IFAT)	[33]
New York	1974	M 38 years	Cyprus 4 years	F 42 years	35 days (1 month)	Recovery	RBCs, PLTs, FFP	*P. malariae*	LM, IFAT (IFAT)	[33]
Tennessee	1974	M 28 years	Nigeria 8 years	F 15 years	3 months (12 days)	Recovery	WB	*P. malariae*	LM (LM, IFAT)	[34]
Wisconsin	1977	N/A	Africa recent	F 57 years	Multiple transfusions (28 days)	Death due to refractory leukaemia	PLTs	*P. falciparum*	LM (LM)	[35]
New York state	1978	M	Ghana 10 months	F 65 years	16 days (1 day)	Recovery	WB, RBCs, FFP	*P. falciparum*	LM (IFAT)	[36]
California	1980	M	N/A	M 6 years	15 days (2 days)	Recovery	RBCs	*P. falciparum*	LM (LM)	[37]
California	1982	M	Nigeria 7 years	M premature	6 weeks	Death due to pneumonia	WB	*P. ovale*	LM, IFAT (IFAT)	[38]
Boston	1982	N/A	N/A	M premature	7 weeks (on the day)	Recovery	RBCs	*P. malariae*	LM (LM)	[39]
Boston	1982	N/A	N/A	M premature	10 weeks (on the day)	Recovery	RBCs	*P. malariae*	LM (LM)	[39]
California	1983	M	Guatemala 6 months	M premature	5 weeks (on the day)	Recovery	WB	*P. vivax*	LM (LM)	[40]
California	1983	M	South America 6 months	M infant	14 days (on the day)	Recovery	WB	*P. vivax*	LM (IFAT)	[40]
Texas	1992	M 19 years	Nigeria 7 months	F 71 years	7 days (on the day)	N/A	RBCs, PLTs	*P. falciparum*	LM (IFAT)	[41]
Texas	1992	M 19 years	Nigeria 7 months	M 65 years	N/A	N/A	RBCs	*P. falciparum*	LM (IFAT)	[41]
California	1992	M 55 years	China 44 years	M 44 years	7 months (3 months)	Recovery	RBCs	*P. malariae*	LM (IFAT)	[41]
Texas	1994	M	Nigeria recent	F 59 years	20 days (on the day)	Recovery	RBCs	*P. falciparum*	LM (LM, IFAT)	[42]
Texas	1994	M	Ghana recent	M 46 years	16 days (7 days)	Recovery	RBCs, FFP	*P. falciparum*	LM (LM, IFAT)	[42]
Pennsylvania	1995	M	Nigeria 3 years	F 72 years	Multiple transfusions	Recovery	RBCs	*P. falciparum*	LM (LM, IFAT)	[43]
Missouri	1996	M	West Africa 1 year	M 70 years	15 days (on the day)	Death	RBCs	*P. falciparum*	LM (LM, IFAT, PCR)	[44]
Missouri	1997	M	West Africa 2 years	M 85 years	21 days (on the day)	Death	RBCs	*P. falciparum*	LM (LM, IFAT, PCR)	[44]
Pennsylvania	1998	M	West Africa 2 years	M 49 years	35 days (on the day)	Recovery	RBCs	*P. falciparum*	LM (IFAT, PCR)	[44]
Texas	2003	M	Ghana 2 years	69 years	17 days (3 days)	Recovery	RBCs	*P. falciparum*	LM (LM, PCR, IFAT)	[45]
Texas	2007	M	Nigeria 6 years	F 25 years	Multiple transfusions	Recovery	RBCs	*P. falciparum*	LM (IFAT)	[46]
Washington D.C.	2007	M	West Africa	F	15 days (on the day)	Recovery	RBCs	*P. falciparum*	LM, PCR (LM)	[47]
Washington D.C.	2007	M 27 years	Nigeria 3 years	M 27 years	13–28 days (11 days)	Recovery	RBCs	*P. falciparum*	LM (IFAT, PCR)	[47]
New Jersey	2007	F 30 years	Uganda > 1 year	M 78 years	1 year	Recovery	RBCs	*P. falciparum*	LM (IFAT, PCR)	[47]
N/A	2007	M 21 years	Benin 4 years	F 55 years	1 month	Recovery	RBCs, PLTs, FFP	*P. falciparum*	LM, IFAT, PCR (IFAT, EIA)	[48]
Georgia	2015	M 20 years	Liberia 15 years	M 76 years	6 months (2 days)	Recovery	RBCs, FFP	*P. malariae*	LM, PCR (LM, PCR, ELISA)	[49]
*Colombia*										
Cali	2011	N/A	Rural area 9 months	F Premature	Multiple transfusions (on the day)	Recovery	RBCs	*P. vivax*	LM (PCR)	[50]
*Brasil*										
Sao Paulo	2008	M	Atlantic forest 1 year	N/A	75 days (on the day)	Recovery	RBCs, PLTs, FFP	*P. malariae*	LM (LM, PCR, IFAT)	[51]
*Spain*										
Valencia	1987	N/A	Congo	F 32years	7 days (on the day)	N/A	WB	*P. falciparum*	LM (IFAT)	[52]
Madrid	1997	N/A	Central Africa	F 63 years	3 weeks (4 weeks)	N/A	WB	*P. falciparum*	LM (IFAT)	[53]
Cordoba	2002	N/A	N/A	F 26 years	Multiple transfusions (128 days)	Recovery	WB, RBCs	*P. falciparum*	LM IFAT	[54]
*UK*										
Midlands	1935	M	India 2 years	M 26 years	19 days (5 days)	Recovery	WB	*P. vivax*	LM (LM)	[55]
London	1938	M	Ceylon 12 years	F 3 months	10 weeks (on the day)	Death	WB	*P. malariae*	LM (LM)	[56]
Durham	1946	M	Yemen 7 years	F 18 years	7–8 weeks (10 days)	Recovery	WB	*P. malariae*	LM (LM)	[57]
N/A	1959	M 19 years	Nigeria 1 year	F 41 years	16 days (6 days)	Recovery	WB	*P. falciparum*	LM (LM)	[58]
Oxford	1966	M	Far East 20 years	M 33 years	10 weeks (1 day)	Recovery	WB, FFP	*P. malariae*	LM (LM)	[59]
Buckingmanshire	1967	M	Army returnee	M 51 years	N/A	Recovery	FFP	*P. malariae*	LM (LM)	[60]
Buckingmanshire	1968	M	Africa 18 months	M 49 years	11 days (12 days)	Recovery	WB	*P. falciparum*	LM (LM, IFAT)	[60]
London	1986	M	Africa	F 72 years	13 days (12 days)	N/A	PLTs	*P. falciparum*	LM (LM, IFAT)	[61]
London	1986	M	Ghana	F 81 years	14 days	N/A	WB	*P. falciparum*	LM (IFAT)	[61]
N/A	1994	F	Ghana 1 year	M	15 days (on the day)	N/A	WB	*P. falciparum*	LM (EIA, IFAT)	[5]
N/A	1997	F 19 years	Ghana 3 years	M 62 years	4 days	Death	WB	*P. falciparum*	(EIA, IFAT)	[5]
N/A	2003	F 38 years	Ghana 7 years	M 51 years	N/A	Death	WB	*P. falciparum*	LM (EIA, IFAT)	[5]
*Netherlands*										
Leiden	2011	M 36 years	Africa Costa Rica > 4 years	F 59 years	2 months (on the day)	Recovery	RBCs	*P. malariae*	LM, PCR (LM, IFAT, PCR)	[62]
*Germany*										
Göttingen	1998	N/A	N/A	M 18 months	14 days (9 days)	Recovery	RBCs	*P. falciparum*	LM	[63]
*France*										
Poitiers	1969	M	Portugal 5 months	F	15 days (1 month)	Recovery	WB	*P. malariae*	LM (IFAT)	[64]
Paris	1957	F	Tunisia 27 years	F 32 years	48 days (4 days)	Recovery	WB	*P. vivax*	LM	[65]
Paris	1973	M	Senegal 13 years	M 30 years	14 days (9 days)	Recovery	WB	*P. falciparum*	LM (IFAT)	[65]
Paris	1975	N/A	N/A	F 24 years	15 days (18 days)	Recovery	WB	*Plasmodium*	LM (IFAT)	[66]
Tours	1977	N/A	N/A	F 47 years	15 days (on the day)	Recovery	WB	*P. vivax*	LM	[67]
Rouen	1976	N/A	Senegal	N/A	12 days (10 days)	Death	N/A	*P. falciparum*	(IFAT)	[68]
Rouen	1976	N/A	Ivory Coast	N/A	13 days (6 days)	Death	N/A	*P. falciparum*	(IFAT)	[68]
Rouen	1978	N/A	N/A	N/A	60 days (2 days)	Recovery	N/A	*P. malariae*	(IFAT)	[68]
Nancy	1979	M	Zaire 1 month	F 29 years	15 days (43 days)	Recovery	RBCs	*P. falciparum* *P. malariae*	LM (IFAT)	[69]
Crèteil	1980	M	Central Africa	M infant	2 months (3 days)	Recovery	RBCs, FFP	*P. malariae*	LM	[70]
Aulnay-sous-Bois	1986	N/A	N/A	F 64 years	16 days (on the day)	Recovery	WB	*P. ovale*	LM	[71]
Libourne	1990	M	Comores < 6 months	F 39 years	1 month (on the day)	Recovery	WB	*P. falciparum*	LM	[72]
Le Chesnay	2002	F 19 years	Africa 4 years	M 81 years	13 days (4 days)	Death	RBCs	*P. falciparum*	LM, IFAT, PCR (IFAT, PCR)	[73]
Tourcoing	2013	N/A	Endemic area 3 years	F 75 years	14 days (8 days)	Death	RBCs	*P. falciparum*	LM (IFAT, PCR)	[74]
*Switzerland*										
Zurich	1999	M 30 years	Cameroon 6 years	M 70 years	14 days (22 days)	Death	RBCs, FFP	*P. falciparum*	LM (IFAT, PCR)	[75]
*Austria*										
Wien	1929	M	Endemic area 10 years	N/A	14 days	Recovery	WB	*P. vivax*	LM	[76]
*Italy*										
Liguria	1963	N/A	N/A	M Premature	28–40 days	Recovery	WB	*P. malariae*	LM	[77]
Liguria	1963	N/A	N/A	F 8 years	1–13 days	Recovery	WB	*P. vivax*	LM	[78]
Liguria	1964	N/A	N/A	F 6 years	Multiple transfusions (4 months)	Recovery	WB	*P. vivax*	LM	[78]
Sicily	2005	M	Philippine	F 35 years	Multiple transfusions (4 months)	Recovery	WB	*P. malariae*	LM	[79]
Veneto	2008	N/A	N/A	F 29 years Morocco	Multiple transfusions (2 weeks)	Recovery	RBCs	*P. vivax*	LM	[80]
*Algeria*										
Algiers	1918	M	Greece 1 month	F	15 days (few days)	Recovery	WB	*P. praecox* ^b^	LM (LM)	[13]
*Lebanon*										
Beirut	2007	N/A	N/A	M 28 years	1 ½ months (2 weeks)	Recovery	RBCs	*P. falciparum*	LM	[81]
Beirut	2010	N/A	N/A	F 46 years	1 month (2 days)	Recovery	RBCs	*P. ovale*	LM	[82]
*India*										
Shimla	2006	N/A	N/A	F 47 years	12 days (on the day)	Recovery	WB	*P. falciparum*	LM	[83]
*Korea*										
Taegu, South Corea	2000	M 21 years	Endemic area	M 1 year	15 days (5 days)	Recovery	RBCs, FFP	*P. vivax*	LM (LM, PCR)	[84]
*Thailand*										
Bangkok	2011	M teenager	Endemic area 3 weeks	F 62 years	15 days (on the day)	Recovery	RBCs	*P. knowlesi*	LM, PCR	[85]
*Malaysia*										
Kuala Lumpur	2012	M 26 years	Myanmar 9 months	M 12 years	1 week (on the day)	N/A	WB	*P. vivax*	LM, PCR (PCR)	[86]
Sabah	2015	M 51 years	Endemic area	F 23 years	16 days (on the day)	Recovery	WB	*P. knowlesi*	LM, PCR (LM, PCR)	[87]

*N/A* data not available, *WB* whole blood, *RBCs* red blood cells, *PLTs* platelets, *FFP* fresh frozen plasma, *LM* light microscopy, *ELISA* enzyme-linked immunosorbent assay, *IFAT* indirect immunofluorescent antibody test, *PCR* polymerase chain reaction

^a^Only non-endemic areas of the country if malaria endemic were included

^b^Possible misidentification of *P. falciparum*

The first report of TTM went back to 1911 and the most recent occurred in 2015, both in USA. The age of TTM case reports ranged from premature children to an 85 years old individual. The partitioning of cases in children and adults (≥ 18 years) when age was available resulted in 2 children and 39 adults for *P. falciparum*, 14 children and 12 adults for *P. malariae*, 8 children and 6 adults for *P. vivax*, 1 child and 3 adults for *P. ovale,* and 2 adults for *P. knowlesi*. Female versus male ratio was 1:1 for recipients and 1:6 for donors.

i. *Plasmodium* species. The most common *Plasmodium* species detected in TTM resulted to be *P. falciparum* (45%) and *P. malariae* (30%); *P. vivax*, *P. ovale* were less frequently observed: 16 and 4% respectively; two TTM were caused by *P. knowlesi* (2%), and one by a mixed infection *P. falciparum/P. malariae*. *Plasmodium praecox*, an avian *Plasmodium* species, was described in a case report whose infection was acquired in Greece [13].
ii. Species involved in fatal outcomes. The majority of fatal outcomes (11/45) was indeed caused by *P. falciparum* whilst all the other fatalities occurred in individuals infected by *P. malariae* (2/30) and *P. ovale* (1/4).
iii. Incubation period (IP). Table 2 shows the differences in the mean incubation times for each *Plasmodium* species between TTM and MTM. For all species, the mean incubation time in TTM was longer, but the most relevant difference was observed for *P. malariae* (63.9 vs 34.6 days, p = 0.006).

**Table 2:** Mean values of transfusion-transmitted malaria (TTM) versus mosquito-transmitted malaria (MTM) incubation time in days

Species	TTM (95% CI)	MTM (95% CI)^a^	p value^b^
*P. falciparum*	25.7 (7.4–43.9)	13.1 (7–27)	0.172
*P. malariae*	63.9 (43.5–84.4)	34.8 (27–37)	*0.006*
*P. ovale*	19.0 (11.7–26.3)	13.6 (8–31)	0.118
*P. vivax*	29.3 (12.3–46.2)	13.4 (11–16)	0.060
*P. knowlesi* ^c^	15.5 (9.1–21.9)	10.0 (/)	0.058

*CI* confidence interval

Significance threshold p value <0.05 (in italic)

^a^As reported by Dover and Schultz [9]

^b^Obtained through one sample two-tailed Student’s t test, using the MTM mean value for the null hypothesis

^c^A range of the mean incubation time for this species in humans was not available in literature, so a direct comparison of CIs was not possible
iv. Blood component causing TTM. The vast majority of TTM cases were caused by whole blood and/or RBCs transfusion; however, two TTM cases due to platelets and one TTM case due to plasma only were reported.
v. Diagnostic method used for screening (if any) and diagnosis. They are also reported in detail in Table 1. Classical Light microscopy (LM) was the diagnostic method used in virtually all cases of TTM. Only in very few cases this was complemented by serology (IFAT: first time in 1974 for a case of *P. malariae* occurred in US, ex-Cyprus) and/or PCR (first time in 1995 for a case of *P. falciparum* occurred in Canada, ex-Mali). Donor “screening” was in fact in the earlier cases the diagnosis subsequently made on the donor, classically with microscopy. Serology (IFAT) was first reported on donors in 1968 (a case of *P. falciparum* occurred in UK, ex-Africa, and a case of the same species occurred in US, ex-Vietnam). When reported, serology (most often IFAT) appears to be by and large the most frequent method used for donor screening.

## Discussion

Transfusion-transmitted malaria is an alternative accidental *Plasmodium* infection which may cause morbidity and mortality especially in non-endemic areas where individuals have no premunition to malaria. Given the long-time span, over a century, of the case reports some countries which were endemic several decades ago are now malaria free such as the case of Greece and Italy. Therefore, it was not possible to infer any particular geographical pattern of TTM, whose occurrence may reflect people movements due to historical events as well as the proximity to a malaria endemic areas; an example is provided by the numerous army returnees from Vietnam to USA in the late 1960s who were not identified at the time as potential malaria infected blood donors, and caused an increase of TTM cases in the following years in USA [9]. Also, a limitation of this systematic review was due to the selection of exclusively case reports in order to describe the main characteristics of each episode; thus, prevalence studies were discarded as well as data on the occurrence of “transfusion outbreaks” such as the 54 cases of *P. vivax* TTM reported by the WHO to have taken place in Spain in 1971 due to a single blood bank in Barcelona [14]. Further limitations are due to the intrinsic nature of a systematic review based on different reports hampering the possibility to ascertain retrospectively how reliable were the clinical history and the timing of the diagnosis for each TTM case. The majority of fatal outcomes (11/45) was indeed caused by *P. falciparum* whilst all the other fatalities occurred in individuals infected by *P. malariae* (2/30) and *P. ovale* (1/4). However, these other fatalities were not attributable to malaria: two deaths were due to pneumonia and one was due to the complications of a premature newborn. Furthermore, all fatalities caused by *P. falciparum* were observed in adults and elderly people, which may reflect other co-morbidities or a more severe prognosis of malaria in adults compared to children within non-immune populations [15].

There are important differences between malaria natural infection and TTM with respect to the incubation time and delayed diagnosis: a longer incubation period was observed for all *Plasmodium* species as reported by Dover and Schultz [9] despite the absence of the pre-erythrocytic phase as the infected blood component directly transmits the erythrocytic stage of the parasite, namely the merozoite, to the recipient. This paradoxical phenomenon might be explained by the small inoculum of parasites from an asymptomatic donor which requires a longer period of time to develop the clinical symptoms [6]. The incubation period of TTM case reports was confirmed to be longer than the one described in natural infections as shown in Table 2: the difference reached statistical significance (p = 0.006) in *P. malariae*, which is arguably the species with the longest incubation time and lowest parasite density. No other statistically significant difference was observed possibly due to the limited number of case reports, thus any interpretation must be taken with caution. Moreover, particularly in some cases of *P. falciparum*, the IP was surprisingly and unusually long, and, although it might explained in theory by an exceedingly small number of parasites inoculated, a reporting error cannot be excluded. Nevertheless, such potential error is expected to have occurred across all TTM cases, thus making the observation still useful to reinforce the need to extend the window of time for a malaria diagnosis in blood transfusion recipients beyond the expected IP. Moreover, according to the reported data none of the TTM cases occurred in individuals with previous history of malaria, thus ruling out the possibility of recrudescence, circulating anti-malarial antibodies (as it would be the case in malaria endemic areas), or prophylaxis which might have delayed the onset of symptoms and diagnosis. Interestingly, the incubation time of the only mixed *P. falciparum* and *P. malariae* infection was of 15 days, a nearly typical incubation time for the dominant *P. falciparum* species compared to the milder *P. malariae* which employs 35 days on average to clinically develop.

Furthermore, the observation that almost half of the TTM cases reported in this systematic review are due to *P. malariae* (N = 30) and *P. vivax* (N = 16) reinforces the need to consider these other *Plasmodium* species as a not negligible cause of transfusion-transmitted malaria aside from *P. falciparum*.

Several layers of complexity underline the risk of TTM in non-endemic areas: on one hand, the limited proportion of potentially infective donors imposes a cost-effective strategy of blood donors screening, on the other hand the accuracy of such screening needs to be optimal for the serious outcomes of TTM in malaria naïve recipients.

In most non-endemic countries the first step in the blood supply chain is an epidemiological questionnaire to assess the potential donor’s risk to be infective which may result in a deferral for two groups of individuals: (i) those who were born and had lived for several years in malaria-endemic areas and (ii) those who were born and are resident in non-endemic areas but had visited an endemic area. According to the European guidelines individuals are acceptable as blood donors when an immunologic or molecular test for malaria is negative after at least 6 months since their last visit to an endemic area. When these donors have resided for more than 3 months in the endemic area, the deferral time may be longer. However long the deferral does not totally exclude infectious semi-immune individuals: in fact cases of TTM have been linked to donations given more than 5 years after the last potential exposure of the donor to *P. falciparum* and several decades in the case of *P. malariae* [3].

## Conclusions

i. The *Plasmodium* species most commonly involved in TTM were, expectedly, *P. falciparum* and *P. malariae*, but cases of *P. vivax* were not infrequent, either. This parasite is not known to remain so long in blood as the two other species, while it shares with *P. ovale* the phenomenon of hepatic hypnozoites (that, however, are not a possible source of transmission before they reach again the bloodstream).
ii. Species involved in fatal outcomes. All fatal outcomes attributable to malaria were caused by *P. falciparum* and none by *P. vivax*, a parasite that has long been considered benign, although its potential to cause severe malaria has been repeatedly demonstrated in recent years [16].
iii. The incubation period was longer than the average IP for mosquito-transmitted malaria, which may be a further reason for lack of suspicion and diagnostic delay.
iv. Almost all TTM cases were caused by whole blood and/or RBCs transfusion, as expected, but for two cases by platelets and one by plasma only.
v. Classical Light microscopy (LM) was used in all cases of TTM for diagnostic purposes. Only in very few cases this was complemented by serology and/or PCR in the more recent period. Serology (IFAT) was the most frequently used method for donor screening.

WHO regulations on blood donation needs to be reinforced as many of the TTM case reports observed even in the time span since blood safety guidelines were implemented could have been prevented if those guidelines had been applied with stringency. Thus, different strategies need to be combined in order to ensure the safety of blood transfusions i.e. blood donor screening by appropriate diagnostic tools, which should probably include molecular tests, and possibly parasite inactivation of the blood supply.

## Abbreviations

CI: confidence interval
ELISA: enzyme-linked immunosorbent assay
EMBASE: Excerpta Medica dataBASE
FFP: fresh frozen plasma
IFAT: indirect immunofluorescent antibody test
LILACS: Latin America and the Caribbean Health Sciences Literature
LM: light microscopy
MeSH: medical subject heading
MMWR: morbidity and mortality weekly report
MTM: mosquito transmitted malaria
PCR: polymerase chain reaction
PLT: platelet
RBC: red blood cell
TTM: transfusion transmitted malaria
USA: United States of America
WB: whole blood
WHO: World Health Organization
